# Impact of Radial Electrode Coverage on the Performance of Liquid-Deployed PMUTs: A Dynamic and Kinematic Study

**DOI:** 10.3390/mi16010080

**Published:** 2025-01-12

**Authors:** Stephen Sammut, Edward Gatt, Ruben Paul Borg

**Affiliations:** 1 Institute of Engineering and Transport, Malta College of Arts, Science and Technology (MCAST), PLA 9032 Paola, Malta; 2 Faculty of ICT, University of Malta, MSD 2080 Msida, Malta; edward.gatt@um.edu.mt; 3 Faculty for the Built Environment, University of Malta, MSD 2080 Msida, Malta; ruben.p.borg@um.edu.mt

**Keywords:** PMUT, ultrasonics, structural health monitoring, MEMS, radial electrode cover, finite element modelling

## Abstract

This paper highlights the optimisation of a key design parameter essential to the development of PMUTs, which are part of the transmitting components of microsensors. These microsensors are designed for use in the Structural Health Monitoring of reinforced concrete structures. Enhancing the effectiveness of the transmitting component allows for greater spacing between microsensors, which in turn reduces the number of devices needed to implement a full structural health monitoring system. PMUTs designed for integration into the pore solution of reinforced concrete structures need to operate effectively with liquid coupling fluids to ensure optimal sonic energy transfer into the structure. This paper outlines the techniques employed to optimize the central electrode’s percentage radial cover of the piezoelectric layer, in circular PMUTs resonating at around 100 kHz. This optimisation was achieved using Finite Element Modelling, laser vibrometry, and hydrophone experimental techniques. The results demonstrated that a radial electrode cover between 65 and 70% significantly enhances the kinematic and dynamic characteristics of a PMUT’s diaphragm when subjected to the excitation of a sine wave electrical signal. The paper also includes advanced time domain finite element analysis, through which the authors aimed to illustrate the diaphragm’s movements at various levels of radial electrode coverage.

## 1. Introduction

Reinforced concrete is a crucial engineering material which is used to construct a variety of civil engineering structures, such as roads, bridges, and residential, commercial, and industrial facilities [1]. Structural Health Monitoring (SHM) of Reinforced Concrete (RC) structures is essential for ensuring their longevity and safety [2,3]. To tackle issues such as chloride ion ingress, SHM can be improved by embedding monitoring devices directly within the structure, thus forming a distributed sensory system [1]. Embedded devices require an efficient communication channel to interact with each other and with devices situated outside the structure. An ultrasonic radiation-based communication channel would be ideal for fulfilling the communication requirements of these embedded sensors [4].

**Figure 1:**
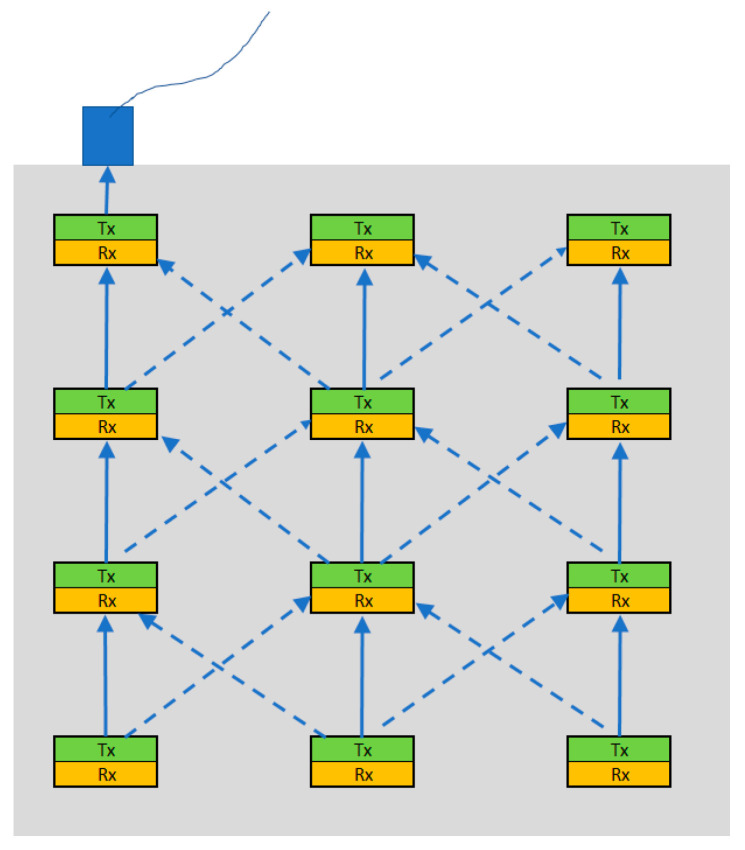
Conceptual setup of a distributed sensory system with the grey shading depicting the concrete structure. The transmitting parts of the devices are shaded in green, while the receiving parts are shown in yellow.

Figure 1 illustrates a concept for a distributed sensor network that could be deployed within a Reinforced Concrete structure. It should be noted that the distributed devices are designed to transmit a signal only when monitored parameters, such as chloride ion concentrations, exceed specific thresholds [4]. This may occur even several years after deployment. The solid blue lines in the figure represent the primary signal path direction, while the dotted lines denote alternative signal paths that offer redundancy. As shown in the figure, the system’s transmitting and receiving components function independently and not as a transceiver, enabling each part to be optimised specifically for transmission or reception.

The inter-device distance between the sensory elements can be increased by enhancing the transmitter’s power or improving the receiver’s sensitivity. Increasing the distance between devices can reduce the deployment costs of the system, as fewer devices would be needed. This paper demonstrates that optimising the electrode radial coverage can enhance the diaphragm’s kinematics, thereby improving the ultrasonic transmission dynamics of the PMUT.

Studies have shown that PMUTs with a diameter of 139 µm and a PZT piezoelectric layer perform optimally with an electrode radial coverage of approximately 66% to 69% when used in liquids [5]. Additional reviewed papers also suggested an optimal electrode coverage of 70%. These studies primarily used air as the coupling fluid [6,7].

Gases typically exhibit low acoustic impedance values, with air having an acoustic impedance of 0.0004 × 1$0^{6}$ kg$m^{-2}s^{-1}$. This value is significantly lower than the acoustic impedance of solids and liquids. Consequently, in finite element modelling, the boundaries between solids or liquids and air are treated as free, behaving similarly to a vacuum [8]. Solids exhibit much higher densities than gasses; however, this value varies widely between different substances. This has an effect on the speed of sound travelling through them with longitudinal wave velocities spanning from 510 m/s in low-density materials like cork to 6100 m/s in metals such as titanium, 7390 m/s in certain stainless steels, and up to 12,800 m/s in beryllium [9]. Hardened concrete exhibits an acoustic impedance of 8.57 × 1$0^{-6}$ kg$m^{-2}s^{-1}$ [10].

Liquids have an acoustic impedance that falls between that of solids and gases. Isopropanol, the liquid coupling fluid used in the experimentation outlined in this paper, has a lower acoustic impedance of 0.912 × 1$0^{6}$ kg$m^{-2}s^{-1}$ [11].

Due to the mismatch in acoustic impedance, a gaseous coupling fluid results in most of the energy which is directed from the PMUT towards the structure being reflected back into the fluid [12]. Therefore, to enable optimal transmission of ultrasonic energy into the structure, a liquid coupling fluid was used rather than a gaseous one. Isopropanol was used as the liquid coupling fluid due to it being unreactive with the PiezoMUMP$s^{TM}$ surface constituents during the experimental processes that were conducted.

The literature review indicated that the optimal frequency for transmitting ultrasonic waves into a reinforced concrete structure was approximately 100 kHz [13]. To achieve resonance at this frequency, the PMUT diaphragm’s diameter needs to be approximately 700 µm or larger [14]. Consequently, this paper focused on PMUTs having diameters of this size. This paper aims to make a significant contribution to the literature in this field by using FEM and experimental methods to identify the optimal radial electrode coverage for achieving the best kinematic performance. The following section will detail the design of the prototype devices.

## 2. The Design

**Figure 2:**
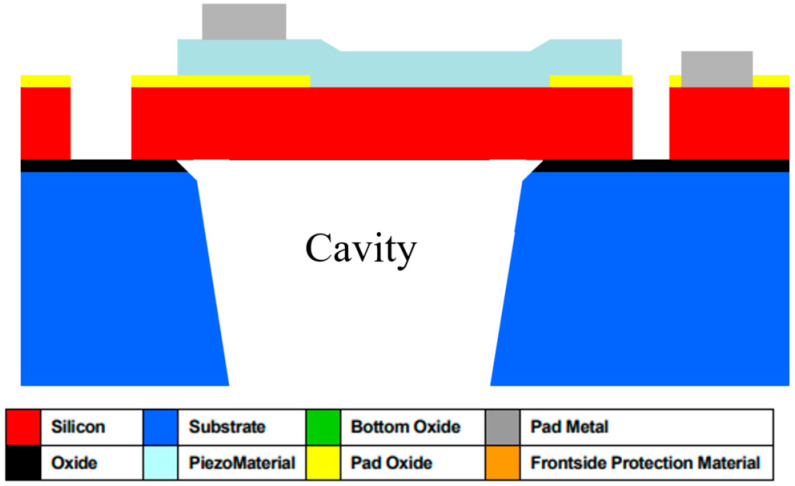
A section through a PiezoMUMP$s^{TM}$ PMUT device.

The devices used in this paper were fabricated using the PiezoMUMPs™ fabrication process for the prototyping of MEMs. Figure 2 shows a cross-section of a PiezoMUMPs™ circular diaphragm PMUT [15].

The PiezoMUMPs™ process employs five distinct masks, each one of which was used to create a different layer of the PMUT’ structure. With reference to Figure 2, the grey-shaded areas constitute the metal layer, which formed the electrodes, tracks, and contacts that electrically connected the PMUT to the external environment. The metal region comprised a 20 nm chrome layer topped with a 1 µm thick aluminium layer. The turquoise-shaded area represents the piezoelectric layer, which generated diaphragm perturbations when electrically stimulated. For this device, the piezoelectric layer was composed of aluminium nitride having a $d_{33}$ strain coefficient of 3.4–6.5 pCN^−1^ [15].

For the purpose of the devices outlined in this paper, the AlN layer was built to cover the full width of the diaphragm, with the metal layer’s radius being varied to achieve different values of radial electrode cover.

Underneath the piezoelectric layer was the pad oxide region, shown in yellow, which acted as a 2000 Å thick insulation layer. Below this, the red-shaded region represents the diaphragm, which was 10 µm thick and made of phosphorus-doped silicon. All these layers were constructed on a 400 µm thick substrate base.

## 3. Finite Element Modelling

**Figure 3:**
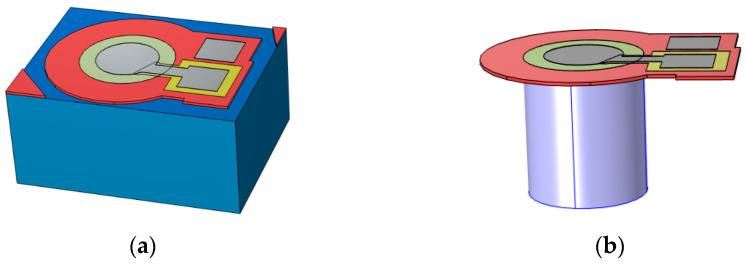
Finite element model showing (**a**) the complete PMUT and (**b**) the cavity fluid area underlying the diaphragm with the substrate removed.

Finite Element Models of the devices were created using COMSOL™ Multiphysics version 6. This software enabled the creation of a multiphysics model, integrating various physical environments into a single simulation. Figure 3 offers a three-dimensional view of the circular diaphragm PMUT model.

Different methods could be employed to optimise electrode configurations, including the analysis of strain mode shapes [16]. However, to achieve the full picture of the underlying physics, full electro–structural–acoustic Finite Element simulations were conducted. These simulations utilised a multiphysics model, which studied the interrelation between three physics interfaces, namely electrostatics, structural mechanics, and acoustics.

**Figure 4:**
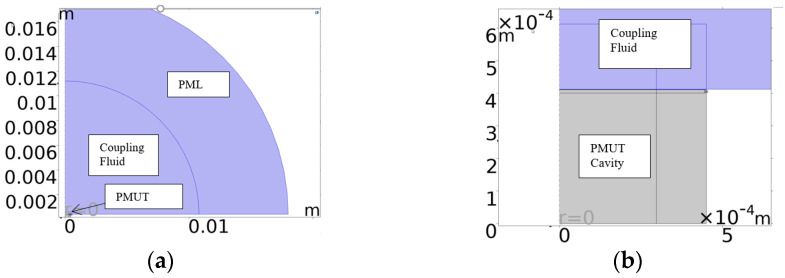
The 2D axisymmetric model showing the (**a**) whole model with a fluid layer over the PMUT, the PML on the outer envelope and the cavity under the PMUT. (**b**) Close-up view of PMUT with PMUT cavity under the diaphragm.

An acoustic structural interface was also set up to model the region in which the diaphragm’s kinetic energy was transferred into the coupling and cavity fluids. Such a modelling strategy is very computationally intensive if used with a 3D modelling platform. To minimise the degrees of freedom required to solve the model, a 2D approach was employed, leveraging the structure’s axisymmetric nature. The axisymmetric model presented in Figure 4 was therefore developed. A Perfectly Matched Layer (PML) was also utilised. This is an artificial absorbing layer placed in the outer envelope of the computational fluid dynamic model which was included as a means of preventing the reflection of waves at the boundaries of the simulation region. 

**Figure 5:**
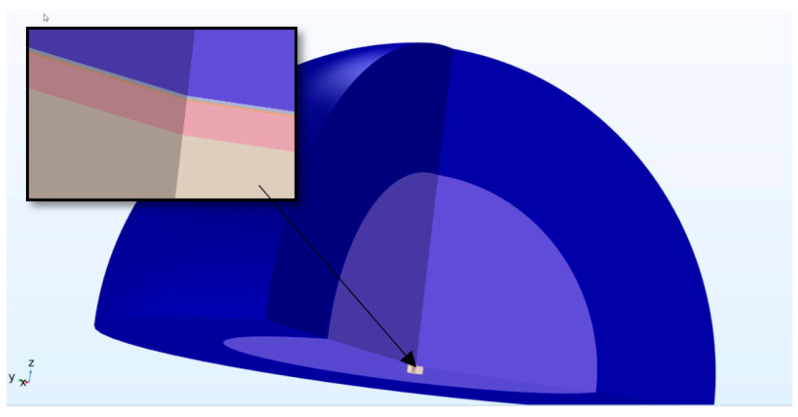
Overview of the axisymmetric model developed in 3D space.

This figure illustrates the diaphragm and the overlying electrode at the midpoint, with the coupling fluid above and the cavity fluid below the diaphragm. The results from the axisymmetric model were further developed by the FEM software, which revolved the 2D image around the axis of symmetry to produce the 3D model shown in Figure 5.

**Figure 6:**
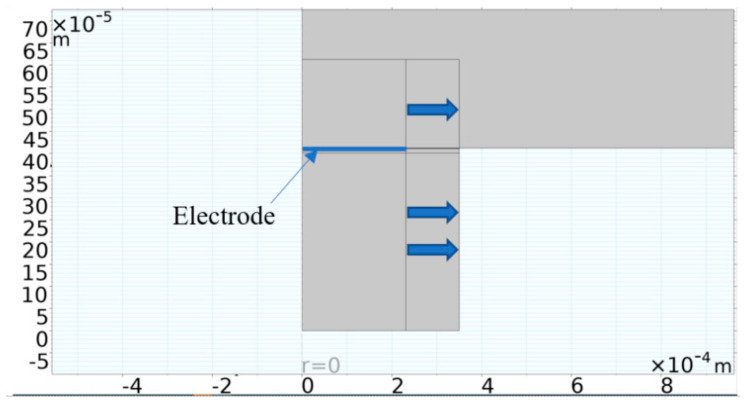
Structure of the FEM used to establish the best percentage electrode radial cover through a parametric sweep ranging between 44% and 99% [14].

Through a parametric sweep, the electrode’s radial cover parameter was stepped in the direction indicated by the blue arrows in Figure 6. The coupling fluid used for the simulation was isopropanol, while the cavity fluid was air.

Frequency domain finite element modelling was conducted on this model, starting with the parametric sweep mentioned above, which incrementally increased the electrode radius, varying the percentage electrode cover parameter between 40% and 99%. 

The insert presented in Figure 5 shows the cavity fluid (lower layer) overlaid by the diaphragm (pink), with the electrode and coupling fluid (light blue) layers shown next. The final layer shown in dark blue is the perfectly matched layer. 

**Figure 7:**
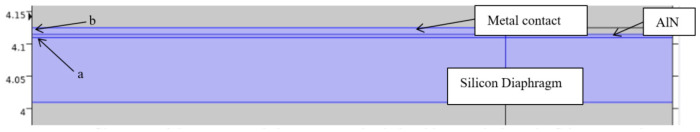
Close-up of the PMUT solid structure (shaded in blue), including the silicon diaphragm, AlN piezoelectric layer, and the Al metal contact. The lower grey area shows the fluid in the cavity, while the upper grey area shows the coupling fluid.

To achieve accurate results, the diaphragm and electrode structures were meticulously designed, as shown in Figure 7. The piezo layer marked *a* in Figure 7, is the sandwich layer between the electrode, marked *b* in the figure and the diaphragm. This piezo layer is not visible in Figure 5 due to its relatively small 0.5 µm thickness.

**Figure 8:**
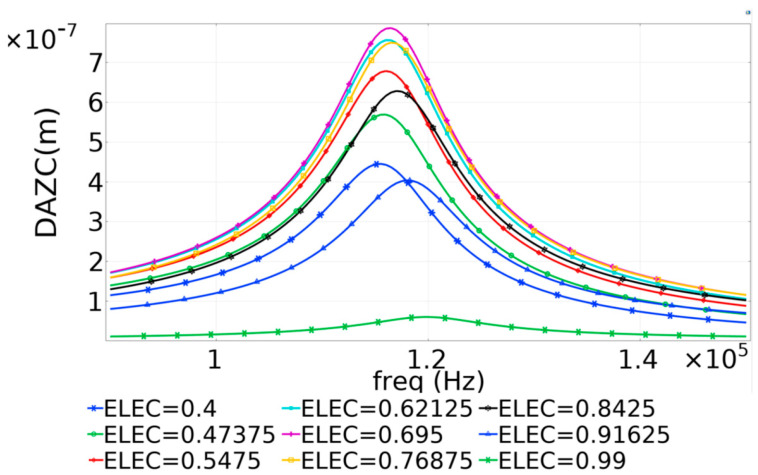
Results of parametric sweep showing the Displacement Amplitude—Z Component (DAZC) for the diaphragm’s midpoint [17].

During the parametric sweep simulation, the frequency was cycled, gradually increasing between 8 × 10^4^ Hz and 1.5 × 10^5^ Hz, in steps of 500 Hz for each value of electrode radial cover. The midpoint displacement values for each frequency step were computed and plotted. The values achieved for the displacement amplitude perpendicular to the diaphragm, the Z component (DAZC) achieved across the entire frequency range are presented in Figure 8.

**Figure 9:**
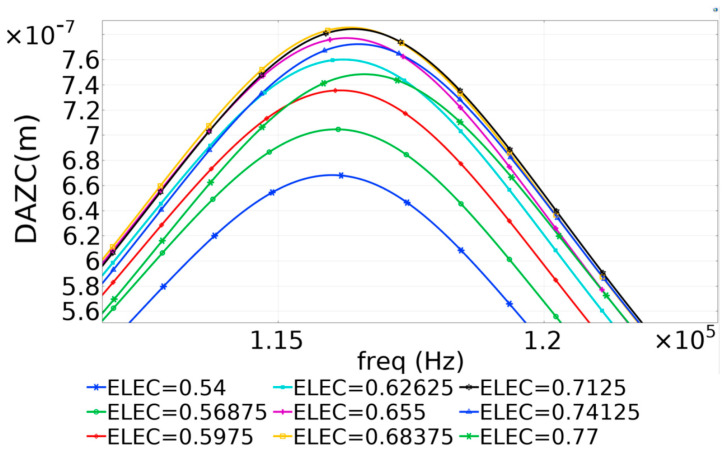
The parametric sweep results for the diaphragm’s midpoint show the Displacement Amplitude in the Z Component (DAZC) [17].

The results presented in Figure 8 were used to identify the resonant frequency for the 700 µm diameter PMUT. Subsequently, the frequency range of the Finite Element Model was narrowed, and the frequency–time steps were shortened to enhance resolution and obtain precise results, thereby achieving the optimal value for radial electrode coverage. The refined plot is shown in Figure 9.

The results indicated that the optimal dynamic performance of the PMUT, as predicted by the Finite Element Method (FEM), was attained when the radial electrode coverage ranged from 65% to 70%. 

**Figure 10:**
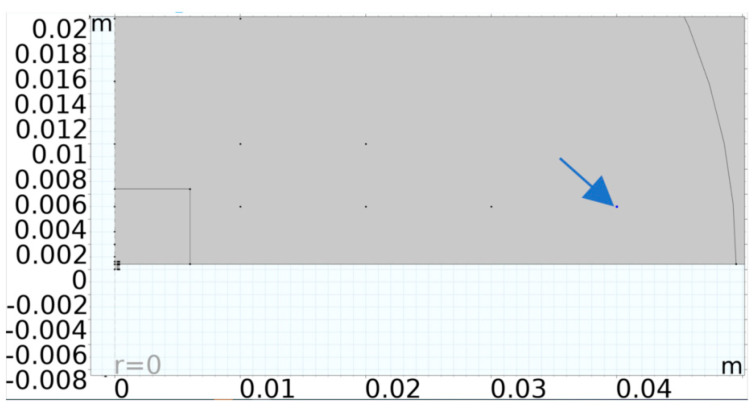
FEM with points at which parameters were recorded. The blue arrow marks the point at which the pressure value being presented in this paper was achieved from the Finite Element Modelling.

Figure 10 shows the measurement points set up in the FEM at which dynamic parameters were measured. Using Finite Element Modelling, the pressure was computed at the point marked by the arrow in Figure 10. This point was 4 cm away from the die. The hydrophone was also positioned at this point during the experimental work outlined in this paper.

The next phase of the project involved validating these results through experimental procedures, which required producing the actual devices for the characterisation process. The following section will describe the device fabrication process in detail.

## 4. Device Fabrication

To validate the Finite Element Modelling results, three prototypes were produced with varying percentages of electrode radial coverage: 98%, 66%, and 50%. The PMUTs with the 98% and 50% values were chosen to thoroughly examine the upper and lower boundaries, respectively. A PMUT with 66% radial electrode coverage was chosen to explore the operating region where the PMUT achieved or approached peak kinematic performance.

The PiezoMUMPs™ process was used to fabricate the devices. This process employed a five-stage lithography technique, with five masks developed for each of the three devices. Table 1 illustrates the processes followed, detailing the steps and masks needed to construct the circular PMUT having 66% radial electrode coverage.

**Figure 11:**
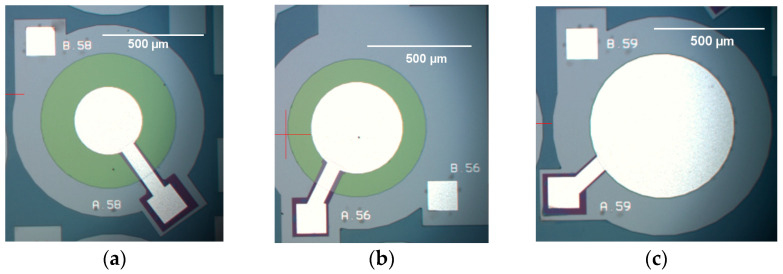
Frontal micrographs of PMUTs, each having a 700 µm diameter cavity with percentage radial electrode coverage of (**a**) 50%, (**b**) 66% and (**c**) 98%.

A similar five-stage lithography process was also used to produce the other two devices, with corresponding masks being designed for each of the PMUTs. The three devices were fabricated on the same die to ensure consistent production conditions, thereby guaranteeing uniform material properties across all three devices. Figure 11 shows the micrographs of the three fabricated PMUTs, each having a different value of radial electrode cover.

The silver-coloured regions indicate the metal layers that formed the contact pads, conducting tracks, and central electrodes, with the latter varying in size as shown. In the exposed areas of devices having 50% and 66% radial electrode coverage, the piezoelectric AlN layer, appearing in green colour, is visible. This is not visible in the PMUT having 98% radial electrode coverage.

The next section of this paper details the device characterisation processes conducted on all three PMUTs.

## 5. Device Characterisation

The device characterisation process was conducted to experimentally confirm the kinematics achieved through Finite Element Modelling. This process involved two experimental stages: one using laser vibrometry and the other utilising an ultrasonic hydrophone.

### 5.1. Laser Vibrometry

Laser Doppler vibrometry was employed to accurately analyse device performance, allowing for the measurement and evaluation of the tiniest diaphragm movements at the nanometre scale. A Polytec MSA 600 laser Doppler vibrometer with a 633 nm wavelength helium–neon source was used for this process since it could measure out-of-plane motion by analysing the frequency and phase shift of the incident laser light that was backscattered by the moving surface [18]. This particular vibrometer, made by Polytec Germany, featured a laser spot size of 1 micron [19]. The small laser spot size, combined with the adjustable angle of incidence, allowed for optimal analysis of both the surface topography as well as the devices’ dynamic response. This setup minimised the effects of reflections and probe needle interference with the laser vibrometer results.

**Figure 12:**
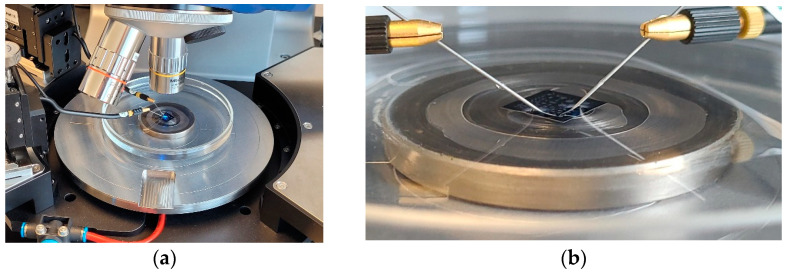
(**a**) Probed die in Petri dish on the laser vibrometer and (**b**) close-up view of the probed devices.

The devices were placed in a Petri dish and submerged in the coupling fluid after being probed, as shown in Figure 12. Probe needles were moved to each device, ensuring that at least one probe needle held down the die at all times to prevent die lift-off, thereby ensuring that the cavity remained air-filled throughout the experimental process.

The Doppler frequency shift of the laser beam was used to measure the velocity along the laser beam’s axis. The PMUT kinematics, as measured by the Laser Doppler Vibrometer, are presented in Table 2.

**Figure 13:**
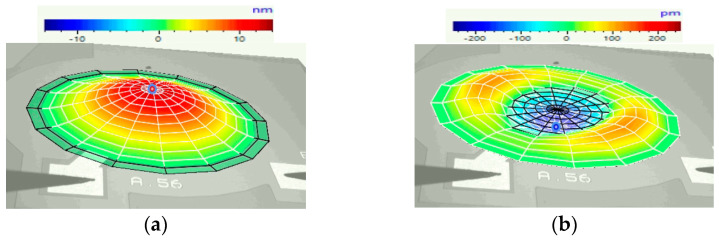
Laser vibrometry results when a device having a 66% radial electrode cover is excited with a periodic chirp signal, showing resonant modes at the excitation frequencies of (**a**) 111.09 kHz and (**b**) 496.88 kHz.

The laser vibrometer characterisation process for each device was conducted in a two-step process. The initial stage utilised a periodic chirp signal and achieved the results shown in Table 2. This was performed to determine the resonant frequency and identify the different modes of vibration. The vibration modes detected for the device with the 66% radial electrode cover are presented in Figure 13. For the purpose of this paper, the resonant frequency that was selected to conduct the work pertains to the fundamental mode.

The next step in the experimental process involved exciting the 66% radial electrode cover PMUT at its resonant frequency using a sine wave excitation signal with a 14 *V_p-p_* amplitude. The laser vibrometer results were normalised to account for the optical effects caused by the liquid coupling fluid on the laser beam. The reason for this was that isopropanol has a different refractive index than air.

**Figure 14:**
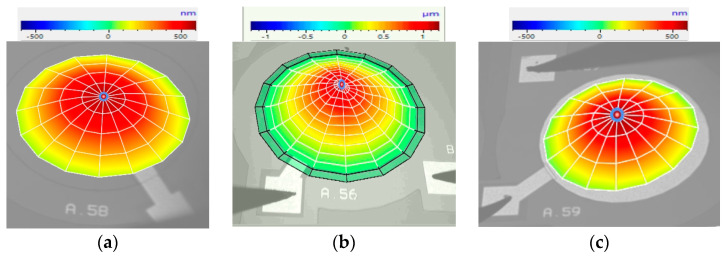
Laser vibrometer results showing peak diaphragm displacement under sine wave excitation for three devices with percentage electrode radial coverage of (**a**) 50%, (**b**) 66% and (**c**) 98%.

This process was also conducted for each of the other devices, with each device being excited at its fundamental mode resonant frequency, as shown in Table 2. Figure 14 displays the laser vibrometer displacement result profile for the three circular PMUT devices, each with different electrode radial cover parameters.

Figure 14 demonstrates that the PMUT with 66% electrode radial coverage achieved the highest displacement levels, as measured by the laser vibrometer and outlined in Table 2. The values shown in Figure 13 and Figure 14 needed to be normalised to account for the impact of the liquid on the laser beam. The results shown in Table 2 were normalised with the coupling fluid’s refractive index, hence the discrepancy of the peak displacement value with the values shown in Figure 14. This normalisation process was carried out using Equation (1) in consultation with the laser vibrometer’s manufacturer [17,20].

Displacement_(actual value in liquid)_ = Displacement_(measured value in liquid)_/refractive-index (1)

### 5.2. Acoustic Characterisation

**Figure 15:**
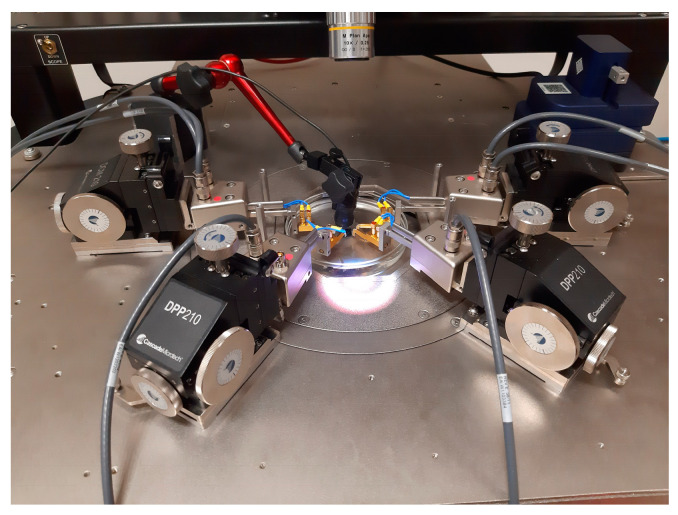
Photo showing the PMUT placed in the Petri dish and the needle probes. The hydrophone is shown attached to the red supporting arm structure [17].

The subsequent stage of the experimental process focused on acoustic characterisation. For this, the device was placed in a 10 cm diameter Petri dish inside a Cascade Probe Station, as illustrated in Figure 15. The device was probed, and the Petri dish was filled with isopropanol to a height of 8 mm. Subsequently, the device was excited through a sine wave signal, and the emitted acoustic signals were captured by a Benthowave BII-7001 hydrophone (Benthowave, Collingwood, ON, Canada), which was positioned at a point 4 cm away from the die, at the same position utilised for the finite element model as shown in Figure 10. Although the hydrophone’s acoustic characterisation process, which was implemented, was deemed to be less precise than laser vibrometry, it effectively measured the pressure in the coupling fluid and was therefore used to validate the pressure dynamics calculated by the Finite Element Modelling.

**Figure 16:**
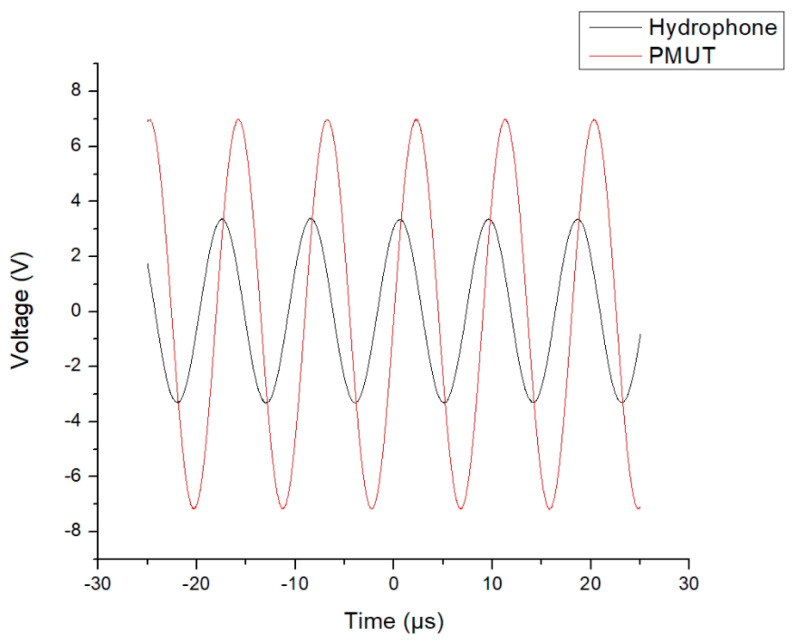
Graph showing the voltage across the PMUT when excited with a signal having a frequency of 110.24 kHz (red curve) and voltage output from the hydrophone amplifier (black curve). The PMUT’s diaphragm’s diameter was 700 µm, over which there was a 66% radial electrode cover.

As stated above, the first device that was acoustically tested was the PMUT, which had a 66% radial electrode cover. The PMUT was excited with a 14 $V_{p-p}$ signal, as plotted in Figure 16. The hydrophone was used to pick up the acoustic emissions, and the voltage emanating from the hydrophone was plotted on the same graph.

The same acoustic characterisation process was then followed for the PMUTs with the 50% and 98% radial electrode cover. The results achieved by the acoustic characterisation process for each of the three devices are presented in Table 3.

This table shows the significantly higher ultrasonic pressure level measured for the 66% radial electrode cover device.

## 6. Discussion

**Figure 17:**
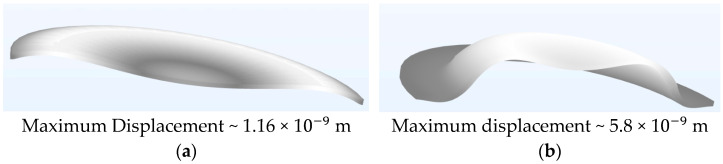
FEM results of PMUT diaphragm with (**a**) percentage electrode radial cover of 98% and (**b**) percentage electrode radial cover of 66%, both taken at the timestamp of 1 × 1$0^{-6}$ s after the initiation of excitation via a 14 $V_{p-p}$ sinusoidal signal. Both diagrams were magnified 15,000 times in the Z direction.

At this stage, time–domain finite element modelling, using the previously detailed axisymmetric model, was conducted to further explore the underlying mechanics of the observed kinematics. In this study, two PMUT models were simulated at their resonant frequency of 110 kHz within a multiphysics environment. One model featured 98% radial electrode coverage, while the other had 66% radial electrode coverage. The simulations also accounted for their coupling and cavity fluids. FEM time stepping was executed in a three-stage approach, with the most precise stage utilising steps of 1 × 10^−7^ s. The small time steps allowed for a detailed visualisation of the PMUT diaphragm’s kinetics. Figure 17 shows the movement of the diaphragm at a timestamp of one microsecond after the start of excitation.

**Figure 18:**
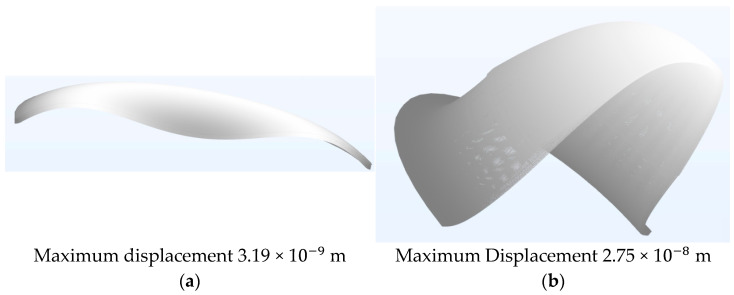
PMUT diaphragm having (**a**) percentage radial electrode cover of 98% and (**b**) percentage radial electrode cover of 66%, both taken at a timestamp of 2 × 1$0^{-6}$ s after the onset of excitation via a 14 $V_{p-p}$ sinusoidal signal. Both diagrams were magnified 15,000 times in the Z direction.

As illustrated in the figure, the Finite Element Model predicted that just one microsecond after the onset of excitation, the maximum displacement reached by the PMUT with a 66% electrode radial cover was already five times that achieved by the PMUT with a 98% radial electrode cover. The difference continued to increase with time, as can be seen in Figure 18, which presents a side-by-side view of the diaphragm shape at a timestamp of 2 × 1$0^{-6}$ s.

The results indicate that the PMUT with a 66% radial electrode cover exhibited a much higher amplitude compared to the device with 98% electrode coverage, even at a timestamp of just 2 microseconds after the onset of excitation. This increase in amplitude lead to greater fluid displacement in the coupling fluid, which in turn generated higher pressures.

**Figure 19:**
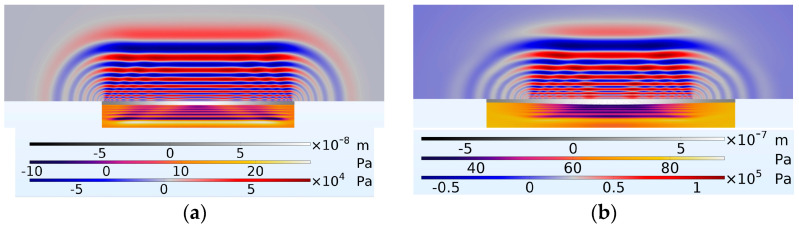
Shows the diaphragm with the coupling fluid layer above and cavity fluid layer below it; (**a**) PMUT with a percentage radial electrode cover of 98% and (**b**) PMUT having a percentage radial electrode cover of 66%. Both shown at a timestamp of 4.002 × $10^{-4}$ s after the onset of excitation.

The next stage of the Finite Element Modelling process was to run the model in a full multiphysics setup. In this way, the electrode/AlN electrostatics physics interface was coupled with the solid mechanics of the PMUT diaphragm, which in turn was coupled with the fluid dynamics of the cavity and coupling fluid regions. The results achieved from the Finite Element Model computation are shown in Figure 19, where the colour code bars from top to bottom refer to the diaphragm’s midpoint displacement, cavity fluid pressure and coupling fluid pressure.

**Figure 20:**
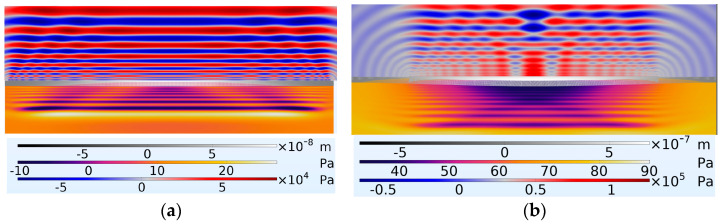
Shows the diaphragm in the middle with the coupling fluid layer above and cavity fluid layer below with (**a**) PMUT having a percentage radial electrode cover of 98% and (**b**) PMUT having a percentage radial electrode cover of 66%. At a timestamp of 4.003 × $10^{-4}$ s after the onset of excitation.

The FEM results in Figure 19 indicate that the PMUT with 66% radial electrode coverage achieved higher coupling fluid pressure levels compared to the PMUT with 98% radial electrode coverage. Even at this initial stage of excitation, the pressure developed in the coupling fluid was already twice as high. This continued to develop, with the pressure difference continuing to increase. Figure 20 presents the pressure waves in the coupling and cavity regions at 4.003 × 1$0^{-4}$ s after the onset of excitation.

In addition to demonstrating an increase in amplitude, Figure 20 reveals that with a 66% radial electrode cover, the ultrasonic radiation was more concentrated towards the diaphragm’s midpoint. This was particularly noticeable when observing the wavefront propagating in the coupling fluid, where the device with 66% electrode coverage showed a higher radiation focus at the midpoint compared to the device with 98% electrode coverage. 

**Figure 21:**
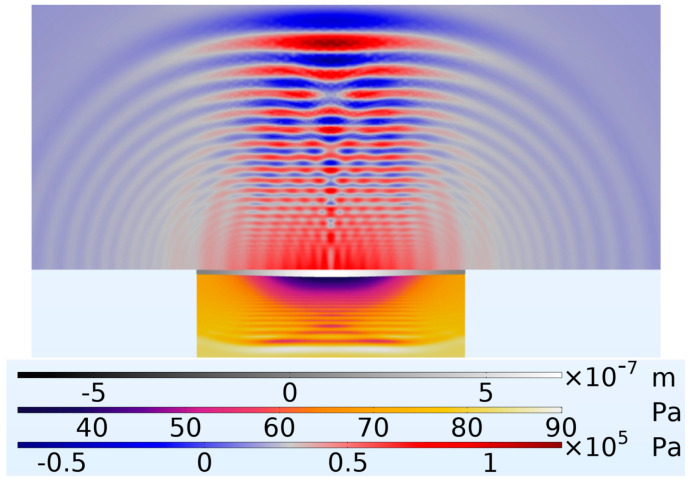
Shows the diaphragm in the middle with the coupling fluid layer above and cavity fluid layer below with a PMUT having a percentage radial electrode cover of 66%. At time t = 4.006 × 10^−4^ s.

Figure 21 presents the pressure waves showing the wave focus in great clarity at a timestamp of 4.006 × 1$0^{-4}$ s after the onset of excitation.

The experimental outcomes from both the laser vibrometer and the hydrophone matched the predictions made by the Finite Element Modelling (FEM). However, there were some deviations from the theoretical predictions. Interestingly, the displacement recorded by the laser vibrometer for the device with 98% radial electrode coverage, while being lower than that for the device with 66% coverage, was still considerably higher in magnitude than the predictions made by the FEM.

## 7. Conclusions

The findings from both the Finite Element Modelling (FEM) and the experimental work which were presented in this paper, both unequivocally confirm that optimising the electrode radial coverage parameter can enhance Piezoelectric Micromachined Ultrasonic Transducer (PMUT) kinematics. The results presented provide a foundation for further research, including the establishment of an enhanced test series with PMUTs of various shapes featuring electrode radial coverage values between 65% and 75%. This, combined with extensive laser vibrometer work, can continue to develop the body of knowledge to enable precise identification of the ideal electrode coverage for all PMUT shapes, designs and deployment solutions.

Another significant aspect is the improved focused pressure output achieved by the PMUT with 66% radial electrode coverage, as shown in Figure 21. This focus is crucial for integrating PMUT devices into array systems, which are useful for achieving beam steering.

Further Finite Element modelling work using powerful computers can also be conducted to further understand the minute details of PMUT kinematic and dynamic interplay. This could essentially include substantial time domain simulations, which are very computationally intensive. Additionally, future research could utilise sophisticated needle probe hydrophones to precisely measure the pressures generated in the coupling fluid by the PMUTs, thus providing valuable feedback for further refinement of the Finite Element Models. These improved models can then be used to develop more advanced PMUT designs.

## Figures and Tables

**Table 1:** Process steps and mask levels used to produce the 66% radial electrode cover circular diaphragm device [15].

Mask Level—Process	Mask	Section Through Device
PADOXIDE—Thermal Oxide	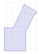	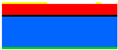
PZFILM—Film Liftoff	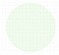	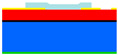
PADMETAL—Padmetal Liftoff	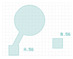	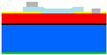
SOI—Silicon Patterning	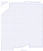	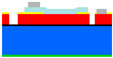
TRENCH—Substrate Patterning	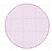	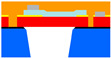

**Table 2:** Comparative analysis of kinematic parameters achieved by laser vibrometry for the three PMUTs. The coupling fluid used was isopropanol, and the cavity was air-filled.

Diameter [µm]	700	700	700
Electrode Radial Cover [%]	98	66	50
Resonant Frequency [kHz]	117.66	111.09	118.75
Displacement ^1^ [µm]	0.64854	0.796	0.42665
Velocity ^1^ [m/s]	0.4794	0.555	0.31834
Acceleration ^1^ [km/$s^{2}$]	354.45	387.39	237.52

^1^: The vibrometer results have been normalised to take into consideration the coupling liquid’s refractive index.

**Table 3:** Comparison of peak pressure measurements in isopropanol coupling fluid for the three PMUTs during acoustic characterisation. All three PMUTs had an air-filled cavity.

Radial Electrode Cover [%]	Frequency [kHz]	Pressure [Pa]
50	108	53
66	110.5	72.25
98	110.8	52

## Data Availability

The original contributions presented in the study are included in the article; further inquiries can be directed to the corresponding author.
